# Amygdala and insula activation in youth with avoidant/restrictive food intake disorder in response to aversive food-specific fear images

**DOI:** 10.1017/S0033291725102766

**Published:** 2025-12-09

**Authors:** Clara O. Sailer, Francesca Galbiati, Laura M. Holsen, Lilian Palmer, Avery L. Van De Water, Thilo Deckersbach, Reitumetse Pulumo, Kendra K. Becker, Lauren Breithaupt, Madison Fisher, Elisa Asanza, Nouchine Hadjikhani, Madhusmita Misra, Kamryn Eddy, Nadia Micali, Elizabeth A. Lawson, Jennifer J. Thomas

**Affiliations:** 1 Neuroendocrine Unit, Department of Medicine, Massachusetts General Hospital, Harvard Medical School, Boston, MA, USA; 2 Multidisciplinary Eating Disorders Research Collaborative, Harvard Medical School, Boston, MA, USA; 3 Division of Endocrinology, Diabetes and Hypertension, Department of Medicine, Brigham and Women’s Hospital, Harvard Medical School, Boston, MA, USA; 4 Division of Endocrinology, Diabetes, and Metabolism, University of California San Francisco, San Francisco, USA; 5 Department of Psychiatry and Division of Women’s Health, Department of Medicine, Brigham and Women’s Hospital, Harvard Medical School, Boston, MA, USA.; 6 University of Applied Sciences, DIPLOMA Hochschule, Bad Sooden-Allendorf, Germany.; 7 Eating Disorders Clinical and Research Program, Massachusetts General Hospital, Harvard Medical School, Boston, MA, USA.; 8 Department of Psychiatry, Harvard Medical School, Boston, MA, USA.; 9 Athinoula A. Martinos Center for Biomedical Imaging, Harvard Medical School, Boston, MA, USA; 10 Psychiatric Neuroimaging Laboratory, Brigham and Women’s Hospital, Boston, MA, USA; 11 Division of Pediatric Endocrinology, Department of Pediatrics, Harvard Medical School, Boston, MA, USA.; 12 Center for Eating and feeding Disorders Research, Mental Health Center Ballerup, Copenhagen University Hospital – Mental Health Services CPH, Denmark; 13 Great Ormond Street Institute of Child Health, University College London, London, United Kingdom

**Keywords:** anxiety, ARFID, feeding and eating disorders, food cues, neuroendocrinology, neuroimaging

## Abstract

**Background:**

Avoidant/restrictive food intake disorder (ARFID) leads to faltering growth and psychosocial impairment. Three phenotypes can co-occur: fear of aversive consequences of eating (*ARFID-fear* phenotype), sensory sensitivity, and lack of interest in eating/food. We hypothesized that youth with ARFID, especially *ARFID-fear* phenotype, would show hyperactivation of fear-related regions in response to ARFID-specific fear images, compared to healthy controls (HC), and activation of these regions would positively correlate with ARFID fear severity.

**Methods:**

Youth (N=103: 76 ARFID, including 20 *ARFID-fear* phenotype; 27 HC) underwent functional MRI scanning while viewing ARFID-specific fear (e.g. vomiting, choking) versus neutral images. We compared blood-oxygen-level-dependent (BOLD) response in fear-related region of interests (ROI; e.g. amygdala, hippocampus, insula) between ARFID and *ARFID-fear* phenotype versus HC. We evaluated the association between brain response and ARFID fear severity in *ARFID-fear* phenotype.

**Results:**

Across individuals, there was a robust bilateral amygdala response to ARFID-specific fear versus neutral images. Compared to HC, *ARFID-fear* phenotype showed a greater insula response to ARFID-specific fear versus neutral images (p=0.049). There were no other group differences and no significant relationships between BOLD response and ARFID fear severity in *ARFID-fear* phenotype.

**Conclusions:**

ARFID-specific fear images elicit amygdala responses across individuals, with greater activation in the insula only in *ARFID-fear* phenotype versus HC. These findings validate the ARFID-specific fear paradigm and highlight the intriguing possibility that, in the *ARFID-fear* phenotype, universally feared experiences such as choking and vomiting serve as the unconditioned stimulus in developing ARFID and may partially be mediated by the insular cortex.

## Introduction

Avoidant/restrictive food intake disorder (ARFID) is characterized by avoidance and restriction in volume and variety of food (American Psychiatric Association, 2022). ARFID often leads to difficulty meeting appropriate nutritional needs, though the body image disturbance that characterizes other eating disorders is not a meaningful clinical driver of food avoidance (American Psychiatric Association, 2022). Three phenotypes have been described: fear of aversive consequences (food avoidance, for example, due to fear of choking, vomiting, anaphylaxis, or gastrointestinal discomfort); sensory sensitivity (avoidance based on the sensory characteristics of food); and lack of interest in food or eating (low interest in or low appetite for food) (American Psychiatric Association, 2022; Thomas et al., 2017). The most broadly used three-dimensional neurobiological model of ARFID describes these three phenotypes along a continuum of severity with possible overlap (Thomas et al., 2017). As treatment options for ARFID are still being developed (Thomas et al., 2021, 2020), improved understanding of the underlying neurobiology could enable the identification of novel treatment targets for different ARFID phenotypes.

Underlying neurobiological mechanisms contributing to the phenotypes of food-specific fear of aversive consequences (hereafter referred to as the *ARFID-fear* phenotype) are especially poorly understood. Many individuals have unpleasant experiences with food at some point in their lives; occasional vomiting and gastrointestinal distress are inevitable. Thus, it is unclear why only a subset of individuals develops a profound fear of aversive consequences and resulting food avoidances that are the hallmarks of the *ARFID-fear* phenotype. Importantly, as cases may present with acute onset following a traumatic event, the *ARFID-fear* phenotype can then manifest with rapid weight loss and possibly refusal to eat solid foods (Thomas et al., 2017). As a result, the *ARFID-fear* phenotype appears to account for the majority of ARFID presentations that progress to the inpatient setting (Bryson et al., 2018), making it a primary driver of severe clinical consequences in ARFID. Current cognitive-behavioral treatment strategies encompass exposure and response prevention with feared stimuli, including both aversive foods and visual or auditory cues associated with the traumatic event (e.g. images or videos of people vomiting or choking) (Thomas & Eddy, 2019).

Amygdala circuits are fundamental to integrate different types of threat-related information and generate anxiety and fear responses (Fox & Shackman, 2019). In humans, amygdala hyperactivity has been linked to hypervigilance in anxiety states, and it has been reported in several disorders characterized by anxiety, including panic disorder, post-traumatic stress disorder, and social anxiety disorder (Etkin & Wager, 2007). In addition, indiscriminate amygdala hyperactivation has been observed across different experimental conditions in generalized anxiety disorder (Akiki et al., 2024; Brandl et al., 2022), confirming the amygdala’s central role in neurocircuits implicated in anxiety and fear. Furthermore, theories behind restrictive eating disorders implicate dysregulation in threat and fear pathways that may contribute to phobic features in individuals vulnerable to the development of ARFID (Thomas et al., 2017). In general, psychophysiological reactivity to fear stimuli may lead to phobic-type anxiety disorders by dysregulation of the defensive motivation system (Lang & McTeague, 2009). The defensive motivation system is characterized by hyperactivation of the amygdala, a region that mediates fear-related endocrine changes (Amaral, Price, Pitkanen, & Carmichael, 1992; Lang & McTeague, 2009). Other brain regions involved in fear modulation are the hippocampus (formation and retrieval of memories related to fear), insula (integration of somatic responses to fear), medial prefrontal cortex (mPFC) (cognitive processing of fear), and anterior cingulate cortex (ACC) (regulation of attention, emotion, and motivation) (Schienle et al., 2005, 2007). To improve our understanding of neurobiological underpinnings in ARFID and especially the *ARFID-fear* phenotype, we designed a functional magnetic resonance imaging (fMRI) paradigm to probe fear related brain areas specific to ARFID-related stimuli. In this paradigm, we compared brain activation in response to ARFID-specific fear images (e.g. people who are choking or having an allergic reaction, images of vomit) to activation in response to neutral images (e.g. household items, people doing neutral activities like reading and using computers).

The overarching aim of this study was to evaluate whether individuals with ARFID in general, and those with the *ARFID-fear* phenotype in particular, show hyperactivation of fear neurocircuitry to disorder-relevant stimuli. First, we validated a novel fear paradigm to ensure our contrasts of interest recruited fear neurocircuitry as intended in each group (i.e. healthy controls (HC), ARFID, and *ARFID-fear* phenotype). Second, we tested the following hypotheses: (i) youth with ARFID, relative to HC, would show hyperactivation of fear neurocircuitry (prespecified regions of interest were amygdala, hippocampus, insula, mPFC, and ACC in response to ARFID-specific fear images); (ii) when analyzed separately, youth with the *ARFID-fear* phenotype would show hyperactivation of fear neurocircuitry (see above) in response to ARFID-specific fear images, compared to HC; and (iii) fear neurocircuitry activation would be positively correlated with severity of ARFID fear symptoms in individuals with the *ARFID-fear* phenotype.

## Methods and materials

### Participants and study design

**Figure 1.**
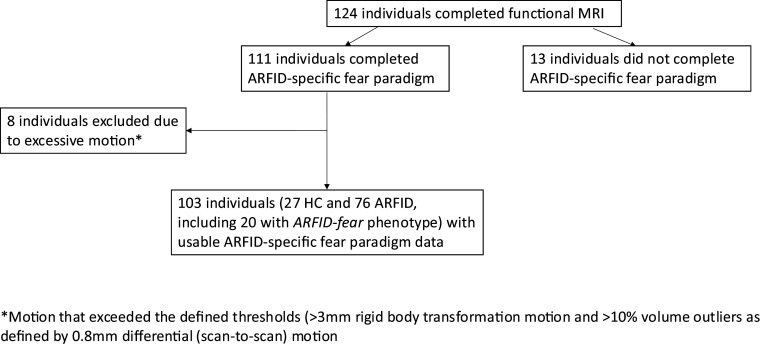
Flow chart summarizing total individuals completed the functional fMRI ARFID-specific fear paradigm, and with usable data for analysis.

We recruited 134 participants for this study: 100 participants with full/subthreshold (see below) ARFID and 34 HC. Of these, 124 individuals completed functional MRI, and 76 with full/subthreshold ARFID, including 20 with the *ARFID-fear* phenotype, and 27 HC had functional MRI data suitable for analysis (Figure 1).

Participants were recruited as part of a multidisciplinary study on the neurobiology of ARFID (R01MH108595) between 2016 and 2021. Participants with full/subthreshold ARFID were eligible if they met criteria for ARFID as determined by the Eating Disorder Assessment for DSM-5 (EDA-5) (Sysko et al., 2015) or if they endorsed significant avoidant/restrictive eating behavior on the Kiddie Schedule for Affective Disorders and Schizophrenia for School Age Children – Present and Lifetime Version 2013 Work Draft (KSADS-PL) (Kaufman et al., 1997). Exclusion criteria for both ARFID and HC included anemia; pregnancy; breastfeeding; use of hormones within the past 8 weeks; history of psychosis; current substance use disorder or current alcohol use disorder assessed using the KSADS-PL; MRI intolerance or contraindications; active suicidal ideation; gastrointestinal tract surgery; any feeding or eating disorder other than ARFID determined by the EDA-5; and past medical history of intellectual disability. Exclusion criteria for HC also included delayed menarche or menstrual irregularity if >2 years post-menarche and a BMI not within the normal range. BMI was considered normal if between the 10-85th percentiles for age and sex for participants younger than 20 years according to the Centers for Disease Control growth charts (Kuczmarski et al., 2002), and if ≥18.5 and < 25.0 kg/m^2^ for participants older than 20 years. We obtained written informed consent from participants age ≥ 18 years. We obtained written informed consent from parents of participants age < 18 years and assent from the participants. All study procedures were approved by the Mass General Brigham Institutional Review Board.

### Procedures

Study visits took place at the Translational and Clinical Research Center and the Athinoula A. Martinos Center for Biomedical Imaging, Massachusetts General Hospital, Boston, MA. At the screening study visit, a nurse practitioner obtained a detailed medical history, a physical exam including height and weight, a blood sample for hematocrit and thyroid function (only if prior history of thyroid disease), and a urine pregnancy test. Eligible participants then returned for the baseline study visit within 8 weeks, where the medical history was updated and the physical exam repeated. Participants were requested to fast overnight and were served a standardized meal prior to the MRI scan (Aulinas et al., 2023). Some cases brought a meal composed of their preferred foods (with prior review and approval by a research dietitian to match the standard meal in terms of calories and macronutrients), and the ARFID-specific fear paradigm (see below for details) took place during an MRI scanning session around 2 hours after the meal.

### Measures

#### Evaluation of ARFID symptoms and assignment to ARFID-fear phenotype

We conferred diagnoses and identified phenotypes of ARFID (versus HC) using the Pica, ARFID, and Rumination Disorder Interview (PARDI), a semi-structured interview developed to diagnose pica, ARFID, and rumination disorder in children and adults (Bryant-Waugh et al., 2019). The PARDI evaluates the presence and severity of the three ARFID phenotypes (i.e. fear of aversive consequences, sensory sensitivity, and lack of interest in eating or food). Example items from the PARDI fear subscale include “Over the past 4 weeks, have you been concerned that eating will make you vomit (i.e., involuntarily)?” and “Over the past month, have you felt afraid of eating?.” Severity and subscale scores range from 0 to 6, with higher scores indicating greater severity. Individuals were categorized as having full ARFID (n = 67) or full *ARFID-fear* phenotype (n = 18) if they met all diagnostic criteria required by the PARDI diagnostic algorithm, and subthreshold ARFID (n = 9) or subthreshold *ARFID-fear* phenotype (n = 2) if they endorsed multiple criteria for ARFID but not at the severity required by the PARDI algorithm (e.g. scoring three out of six for psychosocial impairment, whereas the PARDI requires four out of six for full ARFID). A recent paper presents evidence supporting the reliability and validity of the PARDI in youth with ARFID, proposing cut-off points for identifying the presence of the three phenotypes (Cooper-Vince et al., 2022). However, because the paper specifically suggested that the cut-off point of ≥1.6 for the fear of aversive consequences scale was likely too high and required further validation, we categorized ARFID participants in the current study as belonging to the *ARFID-fear* phenotype based on a cut-off of 0.5. Thus, of the 76 participants with ARFID, 20 participants were categorized as having the *ARFID-fear* phenotype.

#### ARFID-specific fear paradigm

**Figure 2.**
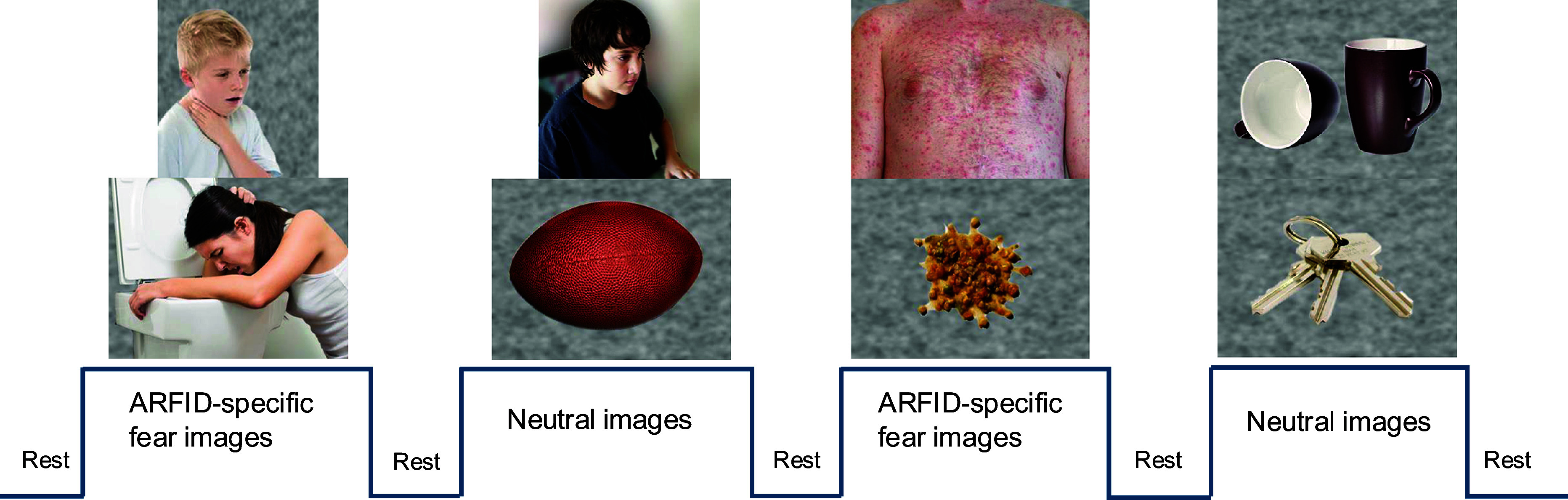
Example images of the ARFID-specific fear paradigm and neuroimaging block design. *Note:* 40 images of each type of stimulus in a block design with the block order pseudorandomized and counterbalanced. Interspersed between blocks of each stimuli type, 10 seconds of fixation was presented. Two 4-minute, 10-second runs (20 images/block; 6 blocks/run) were presented. Full color stimuli were presented for 1.5 seconds; each image was presented twice (once per run), with order of specific images randomized within blocks and across runs.

We created a visual ARFID-specific fear paradigm based conceptually on Schienle’s paradigm (Schienle et al., 2007, 2005) that has been widely applied in imaging and physiological studies of specific phobias (Hermann et al., 2013; Masi et al., 2014; Scharmüller, Wabnegger, & Schienle, 2015). In our version of the ARFID-specific fear paradigm, participants viewed a series of developmentally appropriate images across two categories: ARFID-specific fear images, consisting of eating-related aversive images (e.g. people who are choking, vomiting, or showing an allergic reaction such as hives; images of vomit) and neutral images (e.g. household items such as a basket, a ball, a ladder, a lamp, keys, mugs, and people sitting in front of a computer or writing) (Figure 2). No images contained recognizable food. Prior to the MRI session, participants rated 10 ARFID-specific fear and 10 neutral images on a visual analogue scale indicating how the images made them feel. This assessment was performed to ensure that the ARFID-specific fear images were perceived as more negatively valenced and higher arousal, consistent with the International Affective Picture System for fearful images (Lang et al., 1997). The curser was anchored at 50 (neutral) to allow participants to make an unbiased decision based on how they felt and to change the cursor accordingly: valence scale ranged from 0 (happy, pleased, satisfied, contented, hopeful) to 100 (unhappy, annoyed, unsatisfied, melancholic, despaired, bored), and arousal scale ranged from 0 (stimulated, excited, frenzied, jittery, wide-awake, aroused) to 100 (relaxed, calm, sluggish, dull, sleepy, unaroused). During the MRI session, participants viewed 40 images of each type of stimulus in a block design with the block order pseudorandomized and counterbalanced. Interspersed between blocks of each stimuli type, 10 seconds of fixation was presented. Two 4-minute, 10-second runs (20 images/block; 6 blocks/run) were presented. Full color stimuli were presented for 1.5 seconds; each image was presented twice (once per run), with order of specific images randomized within blocks and across runs. A Dell laptop computer running MATLAB software (MathWorks, Natick, MA) projected visual stimuli onto a screen positioned at the rear of the magnet and viewed via a coil-mounted mirror. Participants were instructed to press a button when pictures changed to ensure attention to the task.

#### Functional MRI data acquisition

Whole-brain imaging was performed on a Siemens 3 T Trio (Siemens, Erlangen, Germany) equipped with a 12-channel head coil. Head movements were restricted with foam cushions. We acquired MRI data using a gradient-echo EPI pulse sequence (33 contiguous oblique-axial slices, 4-mm thick, TR/TE = 2000/30 ms, flip angle = 90°, FOV = 200 × 200 mm, 120 total images per run). A sagittal 3D SPGR (T1-weighted) sequence was acquired (TR/TE = 2350/3.39 ms, flip angle = 7°, FOV = 256 × 256 mm, effective slice thickness = 1.33 mm with 128 slices) for registration of functional datasets.

#### Functional MRI data processing and statistical analysis

We preprocessed and analyzed functional MRI data using Statistical Parametric Mapping v12 (SPM12; Wellcome Department of Cognitive Neurology, London, UK; www.fil.ion.ucl.ac.uk/spm). We realigned, spatially normalized, and smoothed functional images with a 6-mm Gaussian kernel. SPM12 is based on a general linear model approach, including random effects (Friston, Jezzard, & Turner, 1994). Specifically, for each participant, condition effects (ARFID-specific fear images, neutral images, etc.) were estimated at each voxel. We used Artifact Detection Tools (https://www.nitrc.org/projects/artifact_detect/) to detect outliers in global mean image time series (threshold: 3 standard deviation [SD] outside the mean) and motion (1 mm scan-to-scan). Then, in the first-level model, a nuisance regressor was included that censored these outlier timepoints that exceeded thresholds for global signal and motion. We conducted first-level analyses on our contrast of interest (ARFID-specific fear versus neutral images) using linear contrasts and SPM t-maps, which were then submitted to second-level random-effects group analysis.

Following subject-level analysis, the contrast ARFID-specific fear versus neutral images were introduced into group-level designs according to our hypotheses, and group-level contrast maps were generated for each model to test main effects. First, to validate the novel ARFID-specific fear paradigm within each group, we examined neural activation measured as blood-oxygen-level-dependent (BOLD) response to ARFID-specific fear versus neutral images separately in the HC, ARFID, and ARFID-fear groups. Next, to test whether youth with ARFID and the subset of youth with the *ARFID-fear* phenotype (*n* = 20) showed hyperactivation of fear neurocircuitry, we compared difference in neural activation while viewing ARFID-specific fear versus neutral images, measured as BOLD response, between ARFID versus HC and between the *ARFID-fear* phenotype versus HC. Finally, to explore the relationship between neurocircuitry activation and ARFID severity in the subgroup with the *ARFID-fear* phenotype, we ran a regression model that included as regressor of interest the log-transformed PARDI fear subscale (to account for non-normality of data) and the contrast ARFID-specific fear versus neutral images.

In SPM12, for each group-level model, we examined main effects using the small-volume correction approach, restricting voxel-wise analyses to voxels within *a priori* ROIs. Predefined ROIs were amygdala, hippocampus, insula, mPFC, and ACC. The anatomical ROIs were defined using the Automated Anatomical Labeling atlas version 3 (AAL3 [Tzourio-Mazoyer et al., 2002]). Within each ROI, we report clusters that (a) were initially significant at P < 0.05 uncorrected, (b) met or exceeded an extent threshold of k = 5 for the amygdala and k = 20 for all other ROIs, and (c) additionally met the peak-level threshold of p < 0.05, FWE-corrected for the ROI. SVC analyses were run separately within each ROI. For clusters in *a priori* ROIs reaching statistical significance for the above models, parameter estimates were extracted with the REX toolbox for visual display and plotting (Whitfield-Gabrieli, 2009). In addition to hypotheses for *a priori* ROIs, main effects in whole-brain activation to ARFID-specific fear versus neutral images (i.e. not restricted to *a priori* ROIs masks) were examined at a conservative threshold to guard against spurious findings: significant at p < 0.001, uncorrected and met a whole-brain cluster-level threshold of p < 0.05, FWE-corrected.

We performed baseline analysis of clinical data in R Studio (R Core Team, 2023). All descriptive statistics are reported as mean with SD or median with interquartile range pending normality of data and number with percentages unless otherwise noted. We conducted group comparisons using the student’s t-test or Wilcoxon Rank Sum test depending on data distribution (parametric and non-parametric, respectively). We used chi-squared or Fisher’s exact tests to compare categorical demographic variables. We used a generalized linear model to investigate within group differences for valence ratings (comparing ARFID-specific fear versus neutral images).

We performed a power analysis indicating that our sample size had a > 80% power to detect between-group differences of medium effect size (*d* = 0.50) between ARFID and HC using an ROI approach at a significance level of 0.05 (1-sided).

## Results

### Participant characteristics

Participants’ demographic and clinical characteristics are presented in Table 1. Baseline age and sex distribution did not differ between groups. Individuals with the *ARFID-fear* phenotype had a significantly lower BMI z-score (−1.3 vs −0.1; p < 0.001) and a higher PARDI fear of aversive consequences subscale score (1.5 vs 0.0; p < 0.001), compared to HC. Mental health comorbidities of this population have been previously comprehensively described (Kambanis et al., 2020).

**Table 1.** Participants characteristics

	ARFID (*n* = 76)	*ARFID-fear* phenotype (*n* = 20)	HC (*n* = 27)	ARFID versus HC	*ARFID-fear* phenotype versus HC
Age, years (mean [SD])	16.5 (3.9)	15.9 (4.3)	17.8 (4.0)	0.14	0.13
Sex, male (*n* [%])	37 (50)	9 (45)	12 (44)	0.79	1.0
Race (*n* [%])				0.01	0.28
Asian	0 (0)	0 (0)	3 (12)		
Black or African American	1 (1)	0 (0)	1 (4)		
White	71 (93)	19 (95)	19 (76)		
More than One Race	4 (5)	1 (5)	2 (8)		
Hispanic or Latin (*n* [%])	7 (9)	2 (10)	3 (12)	0.99	1.0
BMI, z-score (mean [SD])	−0.5 (1.6)	−1.3 (1.4)	−0.1 (0.8)	0.15	<0.001
BMI, percentile (mean [SD])	38.9 (34.8)	21.8 (27.9)	49.1 (24.7)	0.16	0.001
PARDI severity (median [IQR])	2.3 [1.7, 2.7]	2.6 [2.1, 3.4]	0.2 [0.1, 0.4]	<0.001	<0.001
PARDI sensory sensitivity (median [IQR])	1.2 [0.8, 2.4]	1.2 [0.6, 2.1]	0.0 [0.0, 0.0]	<0.001	<0.001
PARDI lack of interest (median [IQR])	1.6 [0.5, 3.4]	3.3 [2.1, 4.3]	0.0 [0.0, 0.2]	<0.001	<0.001
PARDI fear of aversive consequences (median [IQR])	0.0 [0.0, 0.5]	1.5 [0.9, 2.5]	0.0 [0.0, 0.0]	<0.001	<0.001

*Note*: *ARFID*, avoidant restrictive food intake disorder; *ARFID-fear* phenotype, subgroup of ARFID with phenotype fear of aversive consequences (PARDI fear subscale ≥0.5); *BMI*, body mass index; *HC*, healthy controls; *IQR*, interquartile range; *n*, number; *PARDI*, Pica, ARFID, and Rumination Disorder Interview; *SD*, standard deviation. Descriptive statistics are reported as mean with standard deviation (SD) or median with interquartile range (IQR) according to normality of data and number with percentages. Group comparisons were done using the Student’s t-test or Wilcoxon Rank Sum test for continuous demographic variables according to normality of data, and Chi-squared or Fisher’s exact tests to compare categorical demographic variables.

### Validation of ARFID-specific fear paradigm within each group

As expected, all groups rated ARFID-specific fear compared to neutral images as more negatively valenced (HC: estimated difference 20.0, 95% CI 14.5, 25.5, p < 0.001; ARFID: estimated difference 27, 95% CI 23, 31, p < 0.001; *ARFID-fear* phenotype: estimated difference 29, 95% CI 22, 36, p < 0.001) and higher arousal (HC: estimated difference 17, 95% CI 10, 23, p < 0.001; ARFID: estimated difference 22, 95% CI 17, 26, p < 0.001*; ARFID-fear* phenotype: estimated difference 23, 95% CI 14, 32, p < 0.001). The groups did not differ from one another in their valence ratings, but the ARFID and *ARFID-fear* phenotypes showed higher arousal compared to HC (HC vs ARFID: p = 0.05, HC vs *ARFID-fear* phenotype: p = 0.008).

**Figure 3.**
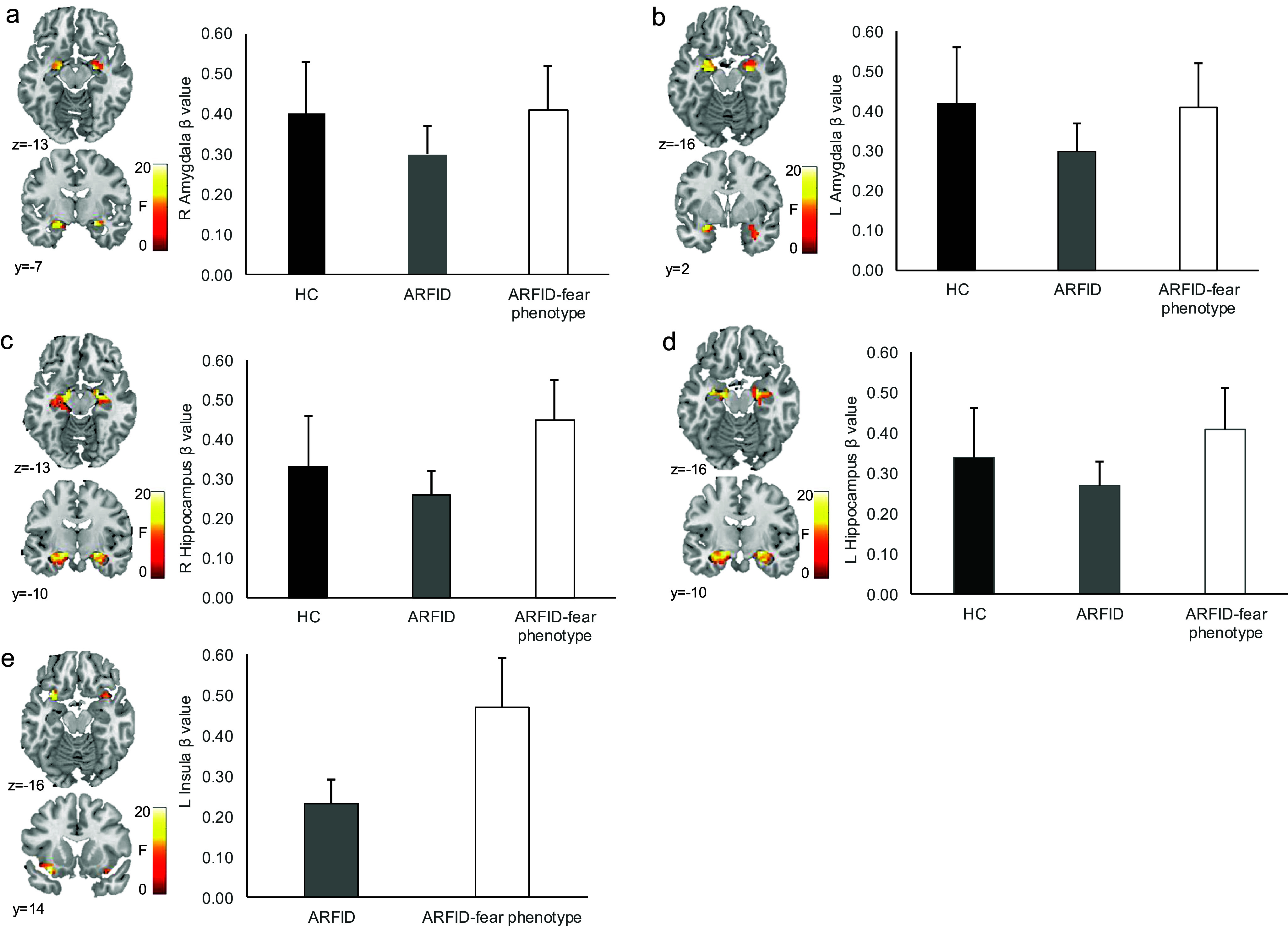
Main effects for HC, ARFID, and *ARFID-fear* phenotype for blood oxygenation level-dependent (BOLD) activation to ARFID-specific fear versus neutral images in *a priori* regions of interest. *Note:* In HC, BOLD activation was significantly increased while viewing ARFID-specific fear versus neutral images in the (a) right amygdala, (b) left amygdala, and (c) right hippocampus. In ARFID, BOLD activation was significantly increased while viewing ARFID-specific fear versus neutral images in the (a) right amygdala, (b) left amygdala, (c) left hippocampus, and (d) left insula. In *the ARFID-fear* phenotype, BOLD activation was significantly increased while viewing ARFID-specific fear versus neutral images in the (a) right amygdala, (b) left amygdala, (c) right hippocampus, (d) left hippocampus, and (e) left insula. Statistical maps for BOLD activation are overlaid on a normalized canonical image (Montreal Neurological Institute, MNI, ICBM 152 nonlinear asymmetric T1 template) with SPM color map corresponding to the relative F value. Coordinates (y, z) are presented in MNI space, with y corresponding to the coronal plane and z to the axial plane. Bar graph depicts mean β values within each cluster for each group and SEM.

In line with our hypotheses, for the contrast ARFID-specific fear versus neutral images, HC exhibited significant BOLD response in the bilateral amygdala (Table 2, Figure 3) and, in the whole brain analysis, in the left angular gyrus and right visual association fields (Table 3).

**Table 2.** *Validation of ARFID-specific fear paradigm* – Main effects for HC, ARFID and *ARFID-fear* phenotype for blood oxygenation level-dependent (BOLD) response to ARFID-specific fear versus neutral images in *a priori* regions of interest

	Hemisphere	Peak t value	K(e)^a^	P (FWE_corr_)^b^	*x* ^c^	*y*	*z*
**HC (*n* = 27)**							
Amygdala	R	4.18	51	**0.012**	24	−4	−16
	L	3.71	53	**0.034**	−27	−1	−22
Hippocampus	R	3.34	39	0.097	21	−4	−19
**ARFID (*n* = 76)**							
Amygdala	R	5.74	64	**0.001**	24	−7	−13
	L	6.56	68	**<0.001**	−21	−4	−16
Hippocampus	L	5.30	52	**0.023**	−18	−7	−13
Insula	L	5.67	100	**0.006**	−36	−4	−13
***ARFID-fear* phenotype (*n* = 20)**							
Amygdala	R	6.61	55	**<0.001**	24	−7	−13
	L	4.15	58	**0.002**	−27	2	−16
Hippocampus	R	5.68	76	**0.005**	24	−10	−13
	L	5.12	99	**0.014**	−18	−10	−16
Insula	L	5.66	106	**0.009**	−33	14	−16

*Note: ARFID*, avoidant restrictive food intake disorder; *ARFID-fear* phenotype, subgroup of ARFID with phenotype fear of aversive consequences (PARDI fear subscale ≥0.5); *HC*, healthy controls; *n*, number; *PARDI*, Pica, ARFID, and Rumination Disorder Interview; *t*, t-statistic.

a Cluster size (contiguous voxels).

b Statistical significance was assessed at *p* < 0.05 FWE-corrected using small-volume correction with a minimum cluster size of *k* > 5 in the anterior cingulate cortex and *k* > 10 in all other regions of interest.

c Coordinates are presented in Montreal Neurological Institute (MNI) space.

**Table 3.** *Validation of* ARFID-specific fear paradigm – Main effects for HC, ARFID and *ARFID-fear* phenotype for blood oxygenation level-dependent (BOLD) activation to ARFID-specific fear versus neutral images at whole brain level

	Hemisphere	Peak *t* value	K(e)^a^	P (FWE_corr_)^b^	*x* ^c^	*y*	*z*
**HC (*n* = 27)**							
Angular gyrus	L	5.88	522	**<0.001**	−54	−67	11
Visual association cortex	R	5.56	518	**0.001**	51	−73	8
**ARFID (*n* = 76)**							
Angular gyrus	L	11.02	1294	**<0.001**	−48	−64	11
Visual association cortex	R	10.84	1629	**<0.001**	51	−67	8
Supramarginal gyrus	L	6.03	167	**0.001**	−57	−37	32
**ARFID-fear phenotype (*n* = 20)**							
Angular gyrus	L	10.7	597	**<0.001**	−48	−61	11

*Note: ARFID*, avoidant restrictive food intake disorder; *ARFID-fear* phenotype, subgroup of ARFID with phenotype fear of aversive consequences (PARDI fear subscale ≥0.5); *HC*, healthy controls; *n*, number; *PARDI*, Pica, ARFID, and Rumination Disorder Interview; *t*, t-statistic.

a Cluster size (contiguous voxels).

b Statistical significance was assessed at *p* < 0.05 FWE-corrected using small-volume correction with a minimum cluster size of *k* > 5 in the anterior cingulate cortex and *k* > 10 in all other regions of interest.

c Coordinates are presented in Montreal Neurological Institute (MNI) space.

Also as expected, in the full/subthreshold ARFID group, for the contrast ARFID-specific fear versus neutral images, we found significant BOLD response in the bilateral amygdala, left hippocampus, and left insula (Table 2, Figure 3). In the whole brain analysis, we found BOLD response in the left angular gyrus, right visual association fields, and left supramarginal gyrus (Table 3).

In line with hypotheses, individuals with the *ARFID-fear* phenotype, for the contrast ARFID-specific fear versus neutral images, showed significant BOLD response in the bilateral amygdala, bilateral hippocampus, and left insula (Table 2, Figure 3). Examination of BOLD response across whole brain yielded identification of significant activation in the left angular gyrus among individuals with *ARFID-fear* phenotype (Table 3).

### Group comparison (ARFID versus HC; ARFID-fear phenotype versus HC) of fear neurocircuitry activation in response to the ARFID-specific fear paradigm

In contrast to hypotheses, in the comparison between ARFID versus HC, there were no significant differences in BOLD response to ARFID-specific fear versus neutral images in *a priori* ROIs or in the whole brain analysis. However, consistent with our hypotheses, among individuals with the *ARFID-fear* phenotype versus HC, we found a greater difference in the BOLD response to ARFID-specific fear versus neutral images in the left insula (p(fwe_corr_) = 0.049, Ke 103, T 4.05, x = −33, y = 14, z = −13) (Figure 3).

### Relationship between fear neurocircuitry activation and ARFID fear severity

In contrast to hypotheses, in the model including ARFID fear severity as covariate, we found no significant clusters in any of the *a priori* ROIs or in the whole brain analysis.

## Discussion

In this novel investigation of brain activity in response to fearful stimuli specifically relevant to ARFID, both full/subthreshold ARFID and those with the *ARFID-fear* phenotype had robust BOLD response in the bilateral amygdala and hippocampus while viewing ARFID-specific fear versus neutral images. A robust activation in the amygdala and hippocampus was also observed among healthy controls, without significant group differences. Individuals with *ARFID-fear* phenotype had greater BOLD response to ARFID-specific fear versus neutral images in the left insula compared to HC, more specifically, in a subregion mapping to the left ventral anterior insula. These findings suggest that these regions play a role in the normative response to ARFID disorder-relevant fear stimuli.

Our results show that all groups rated ARFID-specific fear stimuli as more negatively valenced and higher arousal than neutral images, which indicates that the ARFID-specific fear stimuli lead to a stronger fear response (Lang et al., 1997). However, we did not find hyperactivation in most fear neurocircuitry but a greater ventral anterior insula (VAI) activation in individuals with the ARFID-fear phenotype. The insular cortex is an independent regulator of fear (Klein, Dolensek, Weiand, & Gogolla, 2021) and may mediate specific fear and emotional responses in individuals with an *ARFID-fear* phenotype. The VAI is involved in threat response, and activation of the insula to threat stimuli tracks linearly with fear of pain (Koenen et al., 2017; Sambuco, Costa, Lang, & Bradley, 2020). Among children and adolescents with anxiety disorders, VAI responsivity is associated with behavioral avoidance (Kitt et al., 2024) and is involved in disgust processing (Krolak-Salmon et al., 2003; Woolley et al., 2015). Hyperactivation of the VAI to ARFID-specific stimuli among those with the *ARFID-fear* phenotype may indicate hypersensitivity of the neural fear response in this subgroup, such that maintenance of the anxiety associated with a potential aversive consequence to eating is linked to dysfunctional ongoing over-recruitment of this region during processing of aversive images (Klein et al., 2021).

Furthermore, we found that ARFID-specific fear images increased the amygdala and hippocampus response in individuals with ARFID, *ARFID-fear* phenotype, and HC. Amygdala and hippocampus activation did not differ across groups. Although these findings contrast with our initial hypothesis that ARFID would differ from HC in neural fear response, the lack of differences may be explained by the fact that events such as choking, vomiting, and anaphylaxis are universally distressing to individuals both with and without ARFID. Indeed, the defensive motivation and negative valence systems are highly conserved systems across mammalian species. They represent neurocircuitry responses to aversive situations such as fear, threat, and anxiety, and are deeply involved in social functioning regulation (Hu, Feng, Chen, & Luo, 2023). These neurocircuits are mainly centered in the amygdala, a key region involved in the regulation of both innate and conditioned fear that shows dysregulation in phobic reactions (Garcia, n.d.). Choking and vomiting phobias are specific to ARFID, and restrictive eating is often a consequence of these phobias (Tanldlr & Hergüner, 2015). There is evidence that fear stimuli hyperreactivity distinguishes phobic-type anxiety disorders (Garcia, n.d.) and that specific phobias emerge from classical fear conditioning. Maintenance of these behaviors results in a pattern of learning wherein the phobic stimulus avoidance reduces fear, which, in turn, reinforces the avoidance behavior (Tillfors, 2004). The ARFID-specific fear paradigm used in the current study provides a novel probe into ARFID-related phenotypic behavior given that neuroimaging studies in ARFID so far have focused on assessing brain activation in response to food images (Kerem et al., 2022). Overactivation of the insula, but not of the amygdala, in *ARFID-fear* versus HC may suggest *ARIFD-fear* specific neurocircuits mediating fear processes.

**Figure 4.**
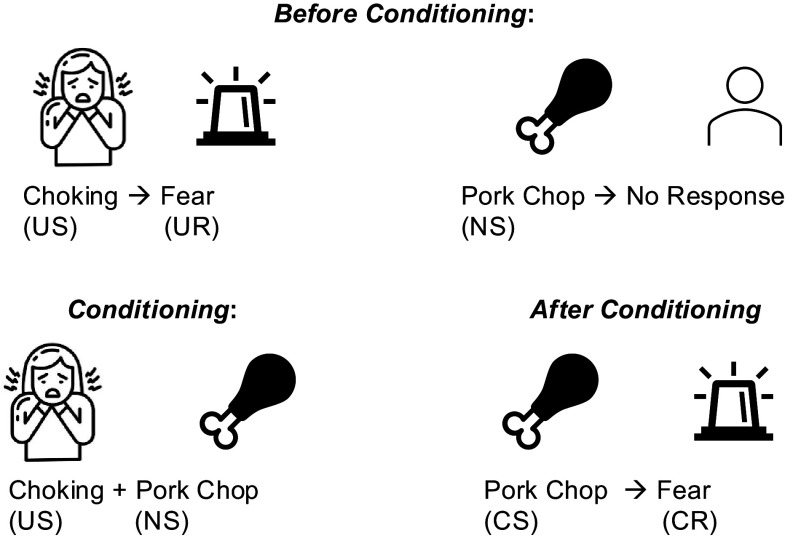
Classic conditioning pathway in ARFID-fear phenotype. *Note:* Classical conditioning theories of the development of specific phobias suggest that individuals come to transfer the fear associated with unconditioned stimulus (i.e. a stimulus that is universally fear-inducing) to a new conditioned stimulus (i.e. an idiosyncratic stimulus that is not universally fear-inducing), and it is this pathological association of fear with the new conditioned stimulus that results in the phobia. In this example of a potential classical conditioning model for the development and maintenance of ARFID-fear phenotype, a young girl exposed to a pork chop (neutral stimulus), develops a specific phobia after choking (unconditioned stimulus), such that the pork chop (now a conditioned stimulus) becomes associated with fear (now a conditioned response).

When combined with our team’s prior findings of *ARFID-fear* phenotype response to food images during functional MRI, the findings of the current study shed light on the possible underlying mechanisms of the *ARFID-fear* phenotype. Specifically, we previously demonstrated that individuals with the *ARFID-fear* phenotype showed significantly greater amygdala activation to images of food (e.g. pizza, salad, sandwiches) versus objects, in comparison to HC (Thomas et al., 2025). Classical conditioning theories of the development of specific phobias suggest that individuals come to transfer the fear associated with an unconditioned stimulus (i.e. a stimulus that is universally fear-inducing) to a new conditioned stimulus (i.e. an idiosyncratic stimulus that is not universally fear-inducing), and it is this pathological association of fear with the new conditioned stimulus that results in the phobia (Figure 4). Figure 4 illlustrates a potential classical conditioning model for the development and maintenance of ARFID-fear phenotype: In this example of a potential classical conditioning model for the development and maintenance of ARFID-fear phenotype, a young girl exposed to a pork chop (neutral stimulus), develops a specific phobia after choking (unconditioned stimulus), such that the pork chop (now a conditioned stimulus) becomes associated with fear (now a conditioned response). Our results are consistent with classical conditioning theory. Specifically, in the current study using a fear image paradigm, all three groups (ARFID, *ARFID-fear* phenotype, and HC) exhibited amygdala activation to vomiting and choking (unconditioned stimuli that are considered universally unpleasant). However, we previously showed that, compared to HC, individuals with the *ARFID-fear* phenotype showed amygdala hyperactivation to images of food (conditioned stimuli that are not considered fear-inducing unless one has ARFID) (Thomas et al., 2025). Future research is needed to further interrogate the role of classical conditioning in the development and maintenance of ARFID, and its potential implications for exposure therapy in ARFID (e.g. consideration of whether food alone should be the exposure target, versus whether stimuli that evoke the potentially aversive consequences associated with eating, such as images or videos of choking or vomiting, must be added to exposures for fear to be extinguished).

Our last hypothesis was that fear neurocircuitry would correlate with ARFID fear severity using the PARDI fear score. However, we did not find such a correlation. This could again reflect the need for identification of specific conditioned stimuli implicated in fear neurocircuitry activation in patients with *ARFID-fear* phenotype. Alternatively, as the PARDI fear cut-off is still being evaluated and prior studies suggested higher cut-offs for severe *ARFID-fear* phenotype, larger studies with different cut-off points might be helpful to improve understanding of *ARFID-fear* phenotype and neurocircuitry activation.

Treatment for the *ARFID-fear* phenotype often focuses on exposure and response prevention to ARFID-specific stimuli (Thomas et al., 2021, 2020). Prior studies have shown that full/subthreshold ARFID elicits a robust neuronal activation to food cues in the amygdala. Our results suggest that the neurobiology of *ARFID-fear* phenotype may be related to both the food itself as well as the consequences of eating (choking, etc.). A potential implication of our findings is that ARFID treatment, particularly for the *ARFID-fear* phenotype, should focus both on decreasing the belief that eating food will inevitably lead to choking/vomiting, and this could be achieved, for instance, with individualized exposure therapy.

Some limitations should be mentioned: fear in full/subthreshold ARFID and especially *ARFID-fear* phenotype is idiosyncratic/individual – someone who is afraid of choking may not be afraid of vomiting, thus limiting our ability to detect neurocircuitry activation to a broad array of fear-inducing images. To improve the specificity of an ARFID-specific fear paradigm, future studies should consider the use of personalized ARFID-specific fear paradigms (e.g. only choking images for *ARFID-fear* phenotype with fear of choking; only vomiting images for *ARFID-fear* phenotype with fear of vomiting). Further, the ARFID-specific fear paradigm is based on the assumption that non-food related imaging would elicit food-related fears in the *ARFID-fear* group, which may not be the case in all participants. Sample size was overall relatively small, especially in the *ARFID-fear* phenotype. Thus, results may be different in a larger sample. It is, however, important to note that ARFID is a rare disease and thus these findings are important to guide future studies. In addition, consistent with clinical practice, participants with ARFID often present with overlapping symptoms of the three phenotypes. The group classified as ARFID-*fear* phenotype expressed fear as a prominent phenotype but not necessarily the only one present. Our cohort was also limited in ethnic/racial diversity in the sample and thus limiting generalizability.

In conclusion, our findings indicate that ARFID-specific fear images elicit robust increases in amygdala and hippocampus activation in ARFID, *ARFID-fear* phenotype, and HC indicative of fear neurocircuitry response and a greater VAI response in the *ARFID-fear* phenotype compared to HC. These findings validate the ARFID-specific fear paradigm and highlight the intriguing possibility that, in the *ARFID-fear* phenotype, universally feared experiences such as choking and vomiting serve as the unconditioned stimulus in the development of ARFID and may be, in part, mediated by the insular cortex.

## Data Availability

Data from this study are publicly available through the National Institute of Mental Health National Data Archive: https://nda.nih.gov.
